# Quantifying depression and the risk of chronic liver diseases: results from a large-scale longitudinal cohort study

**DOI:** 10.3389/fpubh.2025.1584211

**Published:** 2025-09-26

**Authors:** Hailiang Wang, Shuju Zhao, Xueqing Wang, Xiaoyang Liu

**Affiliations:** ^1^Department of Hepatobiliary Surgery, Zibo First Hospital, Zibo, Shandong, China; ^2^Shandong Daizhuang Hospital, Jining, China; ^3^Shengjing Hospital of China Medical University, Shenyang, China; ^4^West China Hospital, Sichuan University, Chengdu, China

**Keywords:** chronic liver diseases, depression, older adults, CHARLS, risk factors

## Abstract

**Background:**

Chronic liver diseases and depression are both major public health concerns worldwide, particularly among aging populations. However, evidence on the prospective association between depressive symptoms and the risk of developing chronic liver diseases remains limited. The aim of this study is to explore the potential association in middle-aged and older Chinese adults.

**Methods:**

Data for this study were obtained from the China Health and Retirement Longitudinal Study (CHARLS), and 11,272 participants without prior liver disease were finally included in this study. Depression was assessed using the CESD-10, with scores analyzed as continuous variables and chronic liver diseases were self-reported based on physician diagnosis. By adjusting for multiple covariates, Cox proportional hazards regression models were used to estimate hazard ratios and restricted cubic spline models were applied to assess the potential non-linear relationships.

**Findings:**

Over a mean follow-up period of 6.85 years, a total of 570 participants were finally diagnosed with chronic liver diseases. Multivariate regression analyses revealed a significant association between CESD scores and the risk of liver diseases among study participants even accounting for all potential covariates (HR: 1.020, 95% CI: 1.006–1.033, *p* = 0.004).

**Conclusion:**

We identified a significant association between depressive symptoms and subsequent development of chronic liver diseases. Based on observational findings, depressive symptoms may represent a potential early marker of liver disease risk. These findings highlight the importance of integrating mental health assessments into early routine clinical care.

## 1 Introduction

Depression is a major global health issue, affecting approximately 350 million people worldwide and having a particularly pronounced impact on older adults (1). Numerous studies have demonstrated that depression adversely affects cognitive function and significantly diminishes quality of life (2). In recent years, increasing evidence suggests that the impact of depression have significantly affected physical health and contributing to the progression of various diseases, particularly within the cardiovascular and digestive systems (3, 4). In middle-aged and older populations, depressive symptoms often coexists with physical comorbidities, exacerbating the risk of chronic diseases and complicating disease management, which highlights the need for deeper insights into how depressive symptoms influence health outcomes in this vulnerable demographic (5).

Liver-related diseases represent a significant and urgent public health issue, particularly in middle-aged and older adults. As life expectancy increases, the burden of non-cancerous liver diseases, such as cirrhosis, non-alcoholic fatty liver disease (NAFLD), and hepatitis, continues to rise (6). Epidemiologically, liver diseases are becoming more prevalent globally, with metabolic and lifestyle-related factors playing major roles in their etiology (7). A better understanding of modifiable risk factors, including mental health conditions like depressive symptoms, is crucial for effective prevention and intervention strategies in this population.

Previous studies have explored the relationship between depressive symptoms and liver diseases, providing preliminary insights but suffering from several limitations. Many studies have relied on cross-sectional designs, limiting the reliability of statistical conclusions and failing to capture the long-term effects of depressive symptoms on liver health (8). Additionally, small sample sizes and selection biases have often restricted the generalizability of findings, and most research has treated the relationship between depressive symptoms and liver disease as linear, without exploring the complex, potentially non-linear dynamics that may exist (9). This study, based on a large-scale, long-term cohort, investigates the association between depressive symptoms and the risk of chronic liver diseases using data from the CHARLS. The objective is to elucidate the complex relationship between depressive symptoms and liver-related diseases and to provide insights that could inform public health strategies for middle-aged and older populations.

## 2 Methods

### 2.1 Data resource and study population

The dataset was sourced from CHARLS, an ongoing cohort study conducted by the National School for Development at Peking University. CHARLS^1^ provides longitudinal data on a wide range of socio-economic and health factors from a nationally representative sample of older Chinese adults. Its conceptual framework and measurement metrics are aligned with those of the Health and Retirement Study (HRS) and other related surveys. A multistage, stratified probability sampling method was employed to ensure a representative sample. The study encompassed 150 municipalities spanning across 28 provinces, municipal communities, and autonomous regions. Beginning with its initial data collection in 2011–2012, CHARLS has continued to gather information on household characteristics, demographics, biomedical measurements and health status every 2 years thereafter. For an in-depth exposition of the survey methodology, please refer to previous publications on the CHARLS survey design (10).

**Figure 1:**
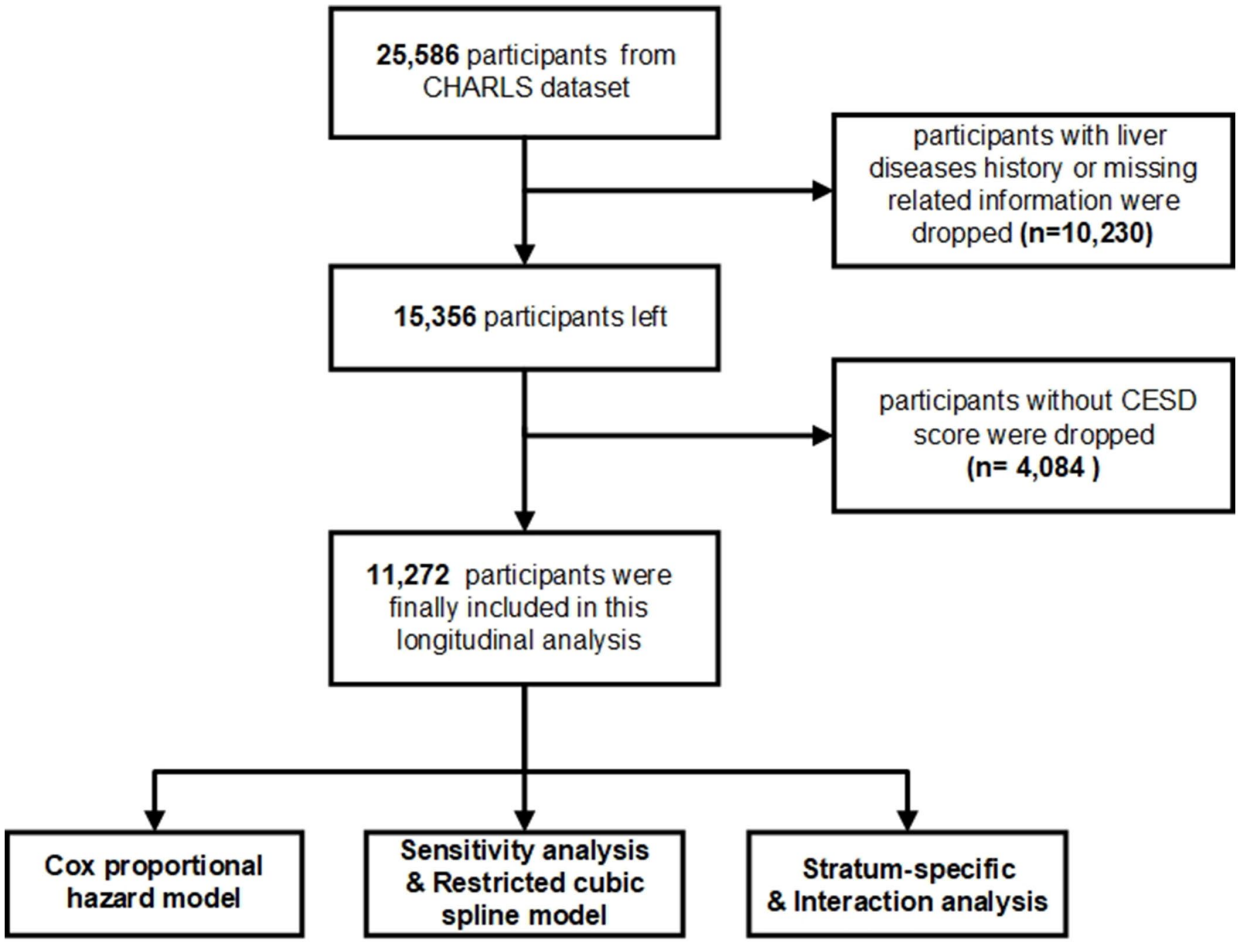
Flowchart of the total participants selection and research design in the CHARLS cohort.

In the current study, a total of 25,586 participants were enrolled were surveyed during CHARLS Wave 1, with 15,356 participants without liver diseases history. Moreover, participants without CESD test were excluded from the analysis were excluded (n = 4,084). Ultimately, a total of 11,272 adults were finally enrolled. Additional information regarding the criteria for participant inclusion and exclusion is detailed in Figure 1.

### 2.2 Definition of depressive symptoms status and liver diseases

The CESD-10, an abbreviated form of the original 20-item CESD, served as a validated screening tool to assess depressive symptoms in the CHARLS cohort. Respondents were asked to rate “how often they felt this way during the past week,” with total scores ranging from 0 to 30. The CESD-10 has been extensively validated for use in general populations and has demonstrated adequate reliability and validity in community-dwelling older adults in China (11).

Chronic liver diseases were identified based on patients’ self-reports regarding a physician’s diagnosis during each visit. The chronic liver diseases include alcoholic liver disease, chronic viral hepatitis, including hepatitis B and C, non-alcoholic fatty liver disease (NAFLD), and hemochromatosis, but except tumors and cancer (12). We defined the respondent as having liver diseases if he answered positively to the query.

### 2.3 Covariates assessment

To reduce the impact of potential confounding variables, this study incorporated specific parameters known to affect the progression of liver diseases. Participants were stratified into distinct groups based on various covariates: education (less than lower secondary, upper secondary and vocational training, and tertiary education); marital status (married or partnered, and separated, divorced, widowed, or never married); residential locations (rural or urban); drinking frequency (never, <1 time/day, 1 time/day, 2 times/day, >2 times/day); smoking frequency (never, <=4 cigarettes, <=10 cigarettes, >10 cigarettes). Moreover, several disorders were identified as potential confounders based on the questionnaire” Have you been diagnosed with listed conditions by a doctor? “The study encompassed preceding medical conditions including hypertension, lung diseases, stroke, psychological disorders, arthritis, kidney disease, gastrointestinal disorders, and asthma. Multivariate imputation techniques were employed to impute missing covariate variables, specifically utilizing predictive mean matching methodologies.

### 2.4 Statistical analysis

For continuous variables that follow a normal distribution, means and standard deviations are utilized as measures of central tendency and dispersion. Conversely, for continuous variables with a skewed distribution, the median and interquartile range are employed. Categorical variables are represented using rates and percentages. The Kruskal–Wallis test was utilized to determine *p*-values for continuous variables exhibiting a skewed distribution, and chi-square tests were employed to analyze categorical data (13).

We calculated hazard ratios (HRs) and 95% confidence intervals (CIs) to analyze the relationship between depressive symptoms and the risk of liver disease. Cox proportional hazards regression models were utilized, with follow-up time employed as the time scale, which was defined from the baseline enrollment visit age until the age when liver diseases were diagnosed, date of death, date of loss to follow-up, whichever event transpired first^2^. Status of depressive symptoms were assessed by using CESD score, which was analyzed as continuous variables, and the proportional hazards assumptions were evaluated using tests based on Schoenfeld residuals. We next performed multivariable Cox Regression models for the main analysis. Specifically, Model I accounted for age; Model II included age, education level, residence, gender, and marital status; Model III further added smoking frequency, drinking frequency, and BMI to the variables in Model II. Additionally, Model IV incorporated underlying diseases based on the variables in Model III. We utilized restricted cubic spline models in conjunction with Cox proportional hazards models to examine potential non-linear relationships, while adjusting for covariates as outlined in crude model. The presence of potential non-linearity was assessed using a likelihood ratio test, comparing the linear model to a model incorporating both linear and cubic spline terms. Subgroup analyses were conducted using Cox proportional hazards models, with the *p*-value for interaction assessed through the log-likelihood ratio test.

In this study, a significance level of 0.05 was employed to determine statistical significance for all findings. All statistical analyses were performed using R version 4.2.2.

## 3 Results

### 3.1 Baseline characteristics

Baseline characteristics stratified by liver diseases are demonstrated in Table 1. The primary analyses included 11,272 participants without liver diseases. Over a mean follow-up period of 6.85 years, a total of 570 participants were finally diagnosed with liver diseases. 1. At baseline, evidence suggests that participants diagnosed with liver diseases exhibit higher levels of depressive symptoms compared to those without liver disease, as indicated by depression scores (9.00 [4.00, 14.00] vs. 7.00 [3.00, 12.00]). Additionally, a higher proportion of participants with liver diseases are male (49.82% vs. 44.86%). Furthermore, the liver disease group has a higher body mass index (BMI) than the non-liver disease group (23.61 [21.39, 26.46] vs. 23.18 [20.93, 25.81]). Statistically significant differences (*p* < 0.001) were also observed in the prevalence of hypertension, diabetes, and chronic conditions such as lung, heart, kidney, and digestive diseases, as well as dyslipidemia, between the two groups.

**Table 1:** Baselines characteristics of the participants enrolled (SD, standard deviation; IQR, interquartile range; BMI, Body Mass Index Mean).

Characteristics	Unit	Liver diseases		*P*
		No	Yes	
		*n* = 10,702	*n* = 570	
Follow-up time (mean ± SD)	Year	6.91 (0.56)	5.97 (1.59)	<0.001
Age (median [IQR])	Year	57.00 [50.00, 64.00]	58.00 [51.00, 63.00]	0.427
Gender (%)	Male	4,778 (44.86)	284 (49.82)	0.023
	Female	5,874 (55.14)	286 (50.18)	
CESD (median [IQR])		7.00 [3.00, 12.00]	9.00 [4.00, 14.00]	<0.001
Age class (%)	<50	2,539 (23.84)	132 (23.16)	0.432
	<60	3,961 (37.19)	202 (35.44)	
	<70	2,934 (27.54)	175 (30.70)	
	> = 70	1,218 (11.43)	61 (10.70)	
Education level (%)	Less than lower secondary	9,551 (89.66)	507 (88.95)	0.725
	Upper secondary and vocational training	970 (9.11)	57 (10.00)	
	Tertiary	131 (1.23)	6 (1.05)	
Marital status (%)	Married or partnered	9,514 (89.32)	513 (90.00)	0.656
	Separated divorced widowed or never married	1,138 (10.68)	57 (10.00)	
Residence (%)	Urban	3,768 (35.37)	209 (36.67)	0.559
	Rural	6,884 (64.63)	361 (63.33)	
Smoking frequency (%)	None	7,478 (75.91)	404 (75.51)	0.418
	<=4	195 (1.98)	16 (2.99)	
	<=10	585 (5.94)	33 (6.17)	
	>10	1,593 (16.17)	82 (15.33)	
Drinking frequency (%)	None	7,217 (71.15)	372 (68.51)	0.626
	Less than once per day	1782 (17.57)	100 (18.42)	
	Once per day	623 (6.14)	39 (7.18)	
	Twice per day	399 (3.93)	23 (4.24)	
	More than twice per day	122 (1.20)	9 (1.66)	
BMI (median [IQR])		23.18 [20.93, 25.81]	23.61 [21.39, 26.46]	0.006
Hypertension (%)	No	8,054 (75.91)	399 (70.25)	0.003
	Yes	2,556 (24.09)	169 (29.75)	
Diabetes (%)	No	10,012 (94.63)	522 (92.23)	0.019
	Yes	568 (5.37)	44 (7.77)	
Lung disease (%)	No	9,787 (92.00)	484 (85.21)	<0.001
	Yes	851 (8.00)	84 (14.79)	
Heart disease (%)	No	9,505 (89.42)	462 (81.20)	<0.001
	Yes	1,125 (10.58)	107 (18.80)	
Stroke (%)	No	10,416 (97.92)	556 (97.72)	0.852
	Yes	221 (2.08)	13 (2.28)	
Psychological fitness (%)	No	10,505 (98.97)	558 (97.89)	0.027
	Yes	109 (1.03)	12 (2.11)	
Arthritis (%)	No	7,189 (67.57)	318 (55.99)	<0.001
	Yes	3,450 (32.43)	250 (44.01)	
Dyslipidemia (%)	No	9,520 (90.88)	472 (83.54)	<0.001
	Yes	955 (9.12)	93 (16.46)	
Kidney disease (%)	No	10,138 (95.43)	498 (88.14)	<0.001
	Yes	486 (4.57)	67 (11.86)	
Digestive disease (%)	No	8,389 (78.88)	392 (68.77)	<0.001
	Yes	2,246 (21.12)	178 (31.23)	
Asthma (%)	No	10,218 (96.22)	527 (92.95)	<0.001
	Yes	401 (3.78)	40 (7.05)	

### 3.2 Association between CESD score and risk of liver diseases

**Figure 2:**
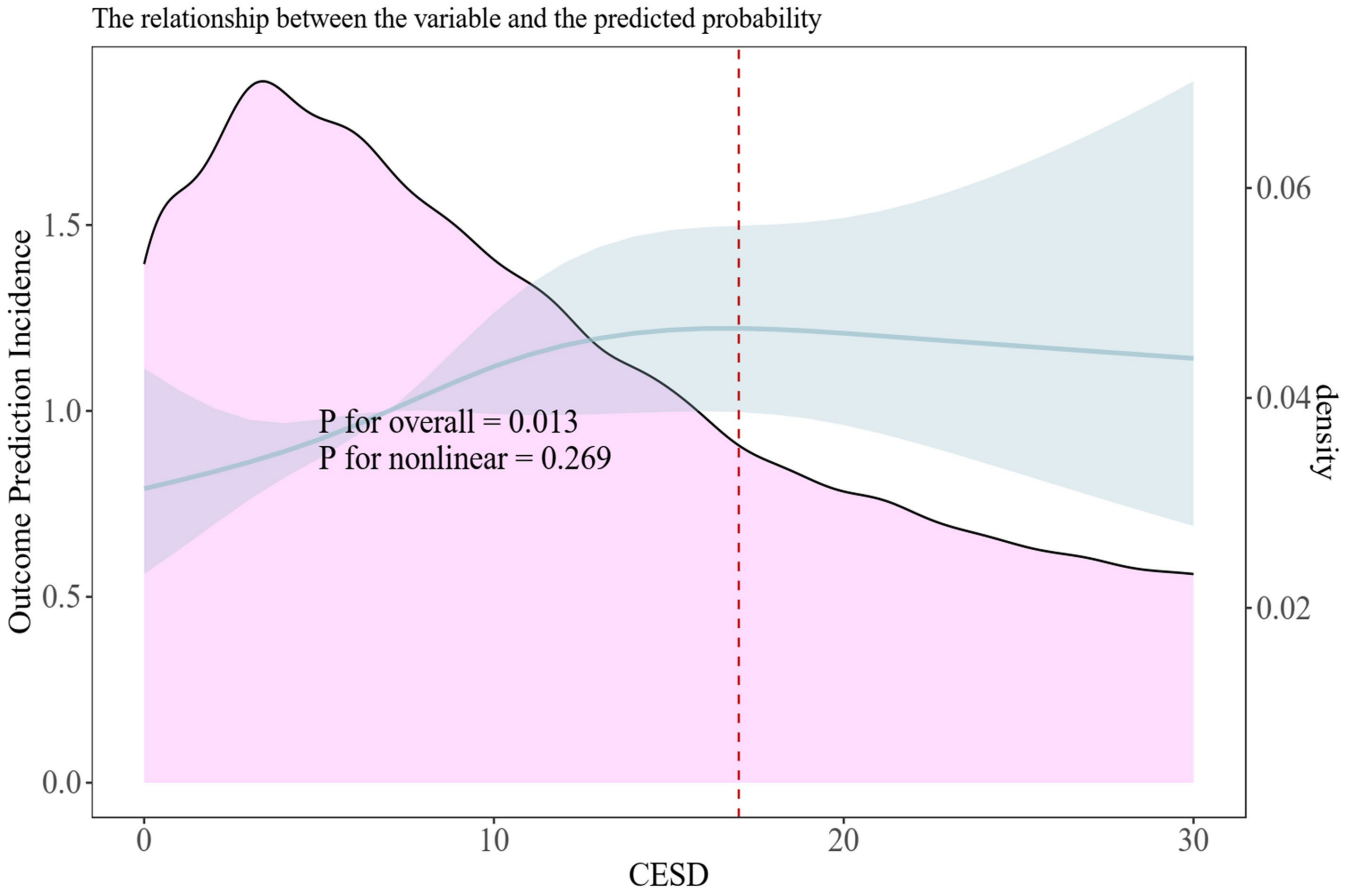
Non-linear associations between CESD score and risk of liver diseases in the CHARLS. Restricted cubic spline models fitted for Cox proportional hazards models for CESD score.

The results of the multivariate regression analyses examining the potential relationship between CESD scores and the risk of chronic liver diseases among study participants are presented in Table 2. In the unadjusted model, higher CESD scores (indicative of more depressive symptoms) were significantly associated with an increased risk of liver disease (HR: 1.033, 95% CI: 1.021–1.046, *p* < 0.001). For Model I, the association remained significant (HR: 1.033, 95% CI: 1.021–1.047, *p* < 0.001). This relationship persisted in Model II (HR: 1.040, 95% CI: 1.028–1.053, *p* < 0.001) and Model III (HR: 1.040, 95% CI: 1.027–1.053, *p* < 0.001). In the fully adjusted Model IV, which incorporated all potential covariates, the association remained statistically significant (HR: 1.020, 95% CI: 1.006–1.033, *p* = 0.004). These findings highlight that comorbidities may affect the observed relationship between depressive symptoms and liver disease risk. Restricted cubic spline models fitted for Cox proportional hazards models for above 4 significant non-linear associations was shown in Figure 2. Relationships were discovered between CESD score and liver diseases (*p* for overall<0.013; p for non-linear = 0.269).

**Table 2:** Hazard ratios (HR) and 95% confidence intervals (CI) for the association between CESD scores and the risk of chronic liver diseases among study participants.

Model	Results	
	HR (95%CI)	*P*-value
Crude model	1.033 (1.021, 1.046)	<0.001
Model I	1.033 (1.021, 1.047)	<0.001
Model II	1.040 (1.028, 1.053)	<0.001
Model III	1.040 (1.027,1.053)	<0.001
Model IV	1.020(1.006,1.033)	0.004

Crude model adjust for none.

Model I adjust for: age.

Model II adjust for: age; gender; education level; marital status; residence.

Model III adjust for: age; gender; education level; marital status; residence; smoking frequency; drinking frequency; BMI.

Model IV adjust for: age; gender; education level; marital status; residence; smoking frequency; drinking frequency; BMI; hypertension; diabetes; lung diseases; heart diseases; stroke; psych problems; arthritis; dyslipidemia; kidney diseases; digest diseases; asthma.

### 3.3 Subgroup analysis

**Figure 3:**
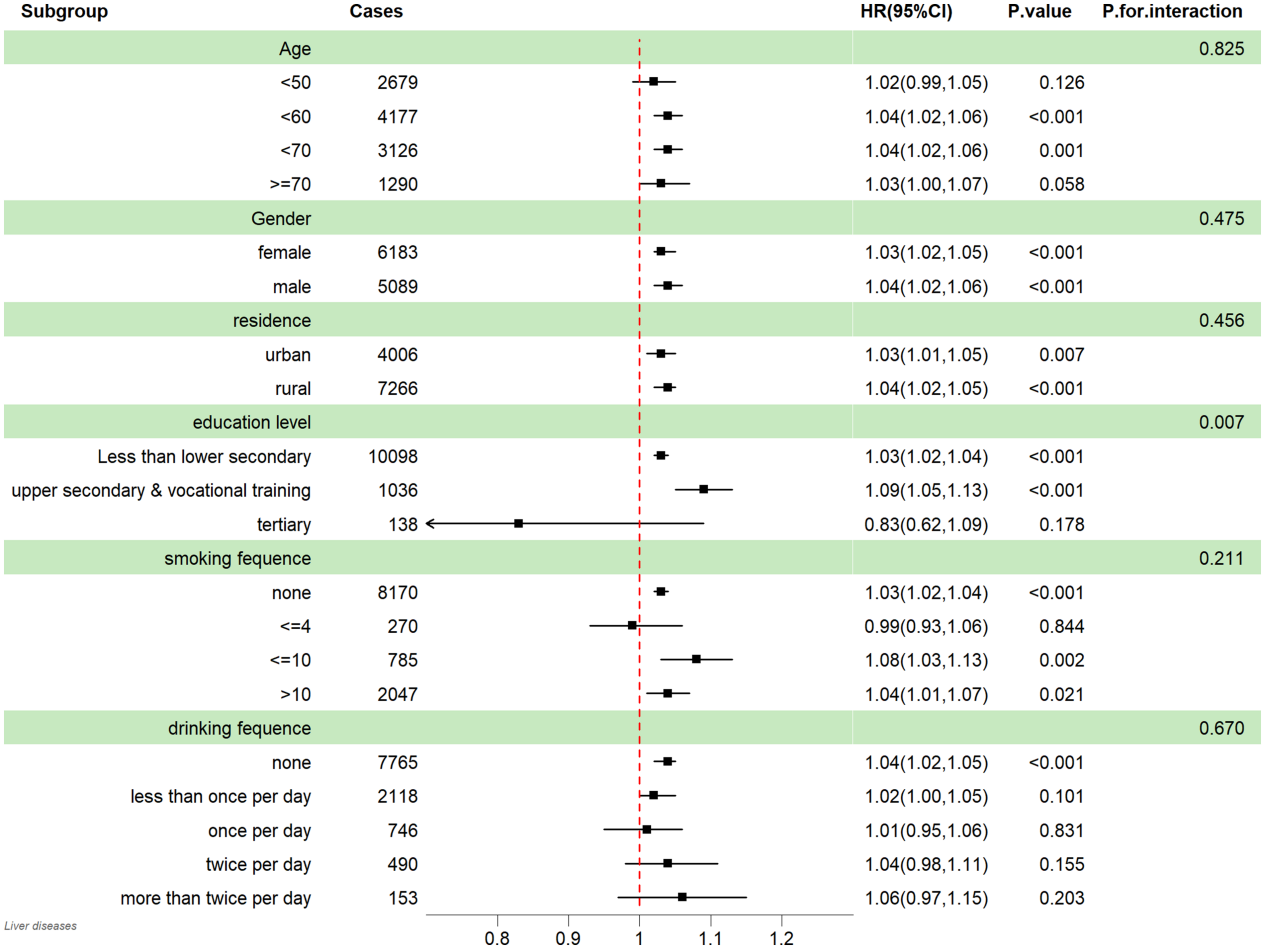
Subgroup analysis between CESD score and liver diseases; HR: hazard ratio; 95% CI: 95% Confidence interval.

Based on the results of Mode IV, the results presented in Figure 3 reveal a noteworthy correlation between CESD score and the probability of experiencing liver diseases among all educational subgroups except for tertiary subgroup. Furthermore, the findings from Figure 3 indicate that this correlation remained consistent across individuals with varying levels of age groups (<50; <60; <70; > = 70); gender (male and female); residence (rural and urban); drinking frequency (never, <1 time/day, 1 time/day, 2 times/day, >2 times/day); smoking frequency (never, <=4 cigarettes, <=10 cigarettes, >10 cigarettes).

## 4 Discussion

Our study reveals a significant positive association between depressive symptoms and the risk of developing chronic liver diseases, which is consistent across different analyses. The robustness of the association was further supported by restricted cubic spline (RCS) curves. This non-linear trend indicates that the impact of depressive symptoms on liver disease risk may be more complex than a simple linear increase.

Previous research exploring the link between depressive symptoms and chronic liver disease has been limited by several key factors (14–16). Many studies have been cross-sectional in design, which compromises the reliability of their statistical findings due to potential reverse causality (16). Moreover, the small sample sizes and selection biases in earlier studies limit their generalizability (14). Importantly, few have considered the possibility of a non-linear relationship between depressive symptoms and liver disease risk (15). Our study addresses this gap, demonstrating that the association may not be a straightforward linear increase but rather an S-shaped curve, indicating that mild to moderate levels of depressive symptoms might have a different risk pattern compared to severe depressive symptoms.

Several biological pathways may explain the observed association between depressive symptoms and chronic liver diseases. Depressive symptoms is known to be associated with dysregulation of the hypothalamic–pituitary–adrenal (HPA) axis, which can lead to increased levels of cortisol and other stress hormones (17–19). This dysregulation has been implicated in promoting liver inflammation and fibrosis, key mechanisms in the pathogenesis of chronic liver diseases (20, 21). Additionally, depressive symptoms is often linked to lifestyle factors such as poor diet, lack of physical activity, and increased alcohol consumption, all of which are known contributors to liver disease (22). Furthermore, depression-related inflammation, marked by elevated levels of cytokines like IL-6 and TNF-α, may contribute to hepatic injury and disease progression (23).

Despite being a large-scale cohort analysis with long-term follow-up to investigate the association between depressive symptoms and chronic liver diseases, there are some limitations. First, our cohort primarily consists of a Chinese population cohort, which may limit the generalizability of our findings to other ethnic groups. Second, detailed information on the liver disease progression and antidepressant use were lacked, influencing the evaluation of potential modifying effect. Third, there was no distinction between different subtypes of chronic liver diseases, which could influence the interpretation of our results. Additionally, the CESD-10 measures depressive symptoms but does not define the clinical diagnosis or severity, which limits further stratified analysis. Finally, self-reported depressive symptoms may introduce reporting bias, despite the use of validated tools like the CESD.

Our findings have important public health implications, particularly for the management of depressive symptoms and chronic liver diseases in middle-aged and older adults. Given the growing burden of both mental health disorders and liver diseases in aging populations, early detection and management of depression could serve as a potential strategy to mitigate liver disease risk. Future research should aim to explore the underlying mechanisms in more depth, particularly focusing on the potential S-shaped relationship between depressive symptoms and liver disease. Additionally, future studies could investigate the impact of depression treatment on liver disease outcomes and assess whether managing depressive symptoms could slow or prevent liver disease progression.

## 5 Conclusion

In this study, we identified a significant association between depressive symptoms and the risk of developing chronic liver diseases. The relationship remained significant after adjusting for all potential covariates and the restricted cubic spline analysis revealed a potential S-shaped curve suggesting that the non-linear relationship. Depressive symptoms may reflect early indicators of increased vulnerability to liver disease, possibly through shared biological or behavioral pathways. These results emphasize the bidirectional and complex interplay between mental and physical health and support the potential value of incorporating mental health assessments into preventive strategies for liver disease. Future research should explore underlying mechanism and integrate mental health assessments into early routine clinical care.

## Data Availability

The original contributions presented in the study are included in the article/supplementary material, further inquiries can be directed to the corresponding authors.

## Footnotes

^1^ https://g2aging.org/

^2^ https://charls.charlsdata.com/pages/Data/harmonized_charls/zh-cn.html
